# 10 years of research on toughness enhancement of structural ceramics by graphene

**DOI:** 10.1098/rsta.2022.0006

**Published:** 2022-09-19

**Authors:** Cristina Ramírez

**Affiliations:** Institute of Ceramics and Glass ICV-CSIC, Kelsen 5, Madrid 28049, Spain

**Keywords:** toughness, graphene, ceramics, reinforcing mechanisms, R-curve, composites

## Abstract

Over the past decade, a new family of ceramic matrix composites has been developed from the incorporation of homogeneously dispersed graphene-based fillers (graphene nanoplatelets/GNP, graphene oxide sheets/rGO or graphene nanoribbons/GNR) into the ceramic matrices. These composites have shown a significant increment of their fracture toughness accompanied by other electrical and thermal functionalities, which make them potentially attractive for a wide range of applications. Here, the main methods for testing the fracture toughness of these composites are described, then the principal observations on the reinforcing mechanisms responsible for this improvement are briefly reviewed, and we discuss the relation with graphene platelets type, morphology and alignment.

This article is part of the theme issue 'Nanocracks in nature and industry'.

## 1. Introduction

Structural ceramics occupy a paramount position in the industrial sector, where parts subjected to high mechanical stress, which operate under high temperature or corrosive environments, are manufactured continuously. The intrinsic brittleness of ceramics, however, limits the practical range of application of these products, creating a need to find strategies to hinder crack propagation from defects and flaws originated at fabrication or during service. The solution to this problem from the composite point of view is a well-known approach in the development of ceramic matrix composites (CMCs) and has been expanded in recent years with the use of graphene-based materials. Since its isolation in 2004, graphene has attracted the attention of the scientific community due to its extraordinary properties as a target material for electronic devices and as a potential filler in composites. The prospects are similar to that of carbon nanotubes (CNTs) in the previous decade. Graphene monolayer exhibits an elastic modulus of 1 TPa and its reported fracture toughness, measured by different methods, is in the range 4–20 MPam^1/2^, depending on sample nature [1]. The first works related to the possible benefits of graphene in the mechanical properties of ceramics date from 2007 [2], when increments in the flexural strength of Si_3_N_4_ composites were reported. Although from the beginning elastic modulus and hardness were shown to not be effectively improved, through observation of indentation cracks, it was noticed that graphene sheets could restrain crack propagation. In 2011, two works, one on Al_2_O_3_/rGO [3] and the other on Si_3_N_4_/rGO [4], demonstrated that important increments in fracture toughness, of 53 and 135%, respectively, could be achieved using homogeneously dispersed graphene sheets, confirming the benefit of using them as structural reinforcement. In the present work, the principal achievements in augmenting the fracture toughness of ceramic composite materials with graphene secondary phases are summarized, using a large number of representative publications about different types of matrices as a reference. Three comprehensive reviews on the topic of graphene/ceramic composites covering their processing methods and properties were published in 2017 [5–7], and because research has incremented significantly in the last five years, most of the referenced papers in this work consider recent publications. The following sections briefly discuss the type of materials that have been developed, the methods for measuring fracture toughness, the maximum increments in *K*_IC_ achieved and the reinforcing mechanisms observed. Complementary research on R-curve behaviour, fatigue and high-temperature properties are also included.

## 2. Materials design and processing methods

Despite the initial difficulties in the mass production of graphene, its availability has increased since 2004, through the discovery of the appropriate conditions for its growth, exfoliation and defects control. Currently, graphene-based materials are produced following several routes, which include mechanical and chemical exfoliation from graphite and other carbon materials, chemical vapour deposition (CVD), epitaxial growth on SiC or molecular assembly [8]. As composite fabrication demands a relatively large amount of material, most of the research has been done by utilizing low-cost exfoliation methods from graphite flakes. Among the most used are ball-milling, sonication in organic media, formation of aqueous suspensions and oxidation, which guarantee high-yield production of the so-called graphene platelets. These structures are stacks of graphene sheets with a thickness from the monolayer to 100 nm [9], and with lateral sizes which depend on the source of graphite and sonication/milling energy and time. Pristine graphene platelets (GNP) exhibit a high tendency to form agglomerates due to restacking during powders mixing by Van der Waals forces, therefore graphene oxide (GO) sheets, highly hydrophilic owing to the functionalized surface, or another derivative such as thermally or chemically reduced graphene oxide (rGO), are more effective at obtaining well-dispersed large and thin graphene platelets, and as discussed in the following sections, also more effective for mechanical reinforcement.

Numerous graphene reinforced-ceramic composites have been studied, with Al_2_O_3_ being one of the main materials used from the beginning [3,10–12] due to the facile processing and its varied industrial uses as refractory ceramic, a substrate for electronic devices or catalytic support. It is followed by Si_3_N_4_ [4,13–17] and SiC [18–21], which are particularly interesting for the fabrication of self-lubricating components exposed to abrasive environments and ZrO_2_ [22–24] for the development of applications in energy and biomedicine. Other matrices include high-temperature-resistant ceramics as B_4_C [25], WC [26], TiC [27], TaC [28], ZrB_2_ [29], TiB_2_ [30], and also materials such as SiO_2_ [31], mullite [32] and hydroxyapatite (HA) [33].

**Figure 1.**
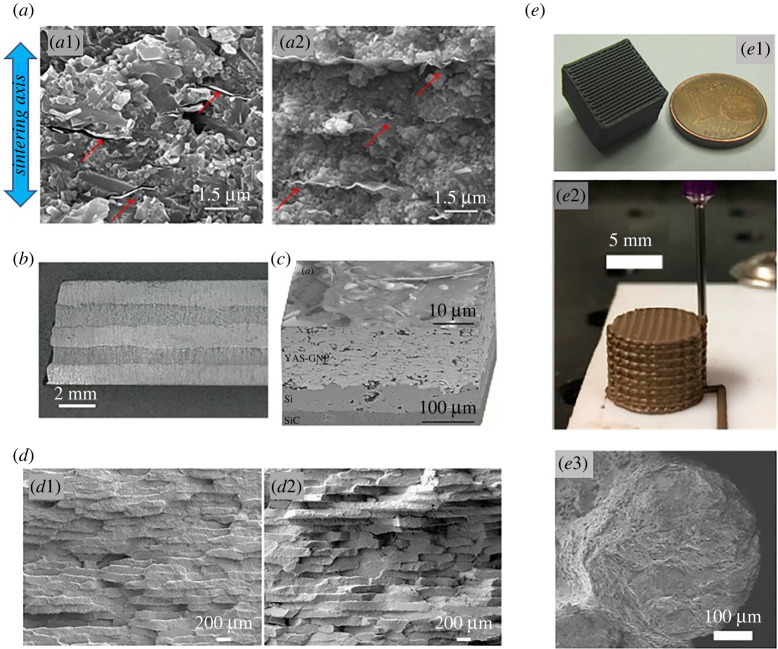
(*a*) SEM images of (*a*1) Si_3_N_4_/GNP and (*a*2) Si_3_N_4_/RGO fracture surfaces showing the typical aspect of the platelets dispersed in a bulk composite, aligned perpendicular to sintering axis due to the uniaxial pressure applied. Adapted from [34] with permission from Elsevier. (*b*) Optical image of a layered composite combining two different contents of graphene. [35] no permission required. (*c*) Micrograph of Y_2_O_3_–Al_2_O_3_–SiO_2_ (YAS)/GNP coating. Reprinted with permission from [36]. Copyright 2015 American Chemical Society. (*d*) SEM micrographs of brick-mortar ZrB_2_/graphene composite with Bouligand structure with (*d*1) 0° and (*d*2) 15° of fibers rotation. Reprinted with permission from [37] Copyright 2020 American Chemical Society. (*e*) Examples of ceramic/graphene three-dimensionally printed structures. (*e*1) SiC/GNP composite scaffold. (*e*2 and *e*3) Al_2_O_3_/GO composite scaffold and detail of rod fracture surface. Adapted from [38], no permission required. (Online version in colour.)

The common composites processing method has been mixing either the GNP or GO/rGO platelets with fine ceramic matrix powders, using powder mixing methods in different media (ethanol, isopropyl alcohol, *N*-methyl-2-pyrrolidone) or colloidal processing, followed by a densification step in which pressure-assisted sintering, like spark plasma sintering and hot pressing, was the main choice. This has been the preferential route for obtaining dense bulk materials, and also produces, as a consequence of the uniaxial pressure applied, anisotropic composites in which graphene platelets *ab*-plane is aligned perpendicular to the pressing direction (figure 1*a*).

Table 1 shows a list of methods used in ceramic/graphene composites processing for dispersion of the graphene fillers. GNP tend to be shorter and more difficult to exfoliate, showing thicknesses usually in the range of 5–20 nm, but thinner sheets can be obtained by the use of specific solvents, such as NMP [10], or milling with additives that increase the stability of dispersion and prevent restacking, like melamine, PEG or PVP [16,39,45]. Conversely, GO is commonly fabricated by Hummers’ method, giving stable aqueous suspensions of platelets with a thickness of less than 5 nm. The functional groups in GO sheets allow the formation of strong bonds with defects present in the matrix and enable GO sheets to interact with positively charged ceramic particles [46]. These conditions create stronger interfaces for a better load transfer [31].

**Table 1.** Examples of graphene exfoliation and dispersion methods used in ceramic/graphene composites processing.

pristine graphene platelets				GO or rGO platelets			
method/solvent	lateral size (µm)	thickness (nm)	ref.	method/solvent	lateral size (µm)	thickness (nm)	ref.
sonication/ethanol-PEG-PVP	0.5–5	0.8–1.2	[39]	Hummers-thermal exfoliation/water	2–3	5	[12]
ball mill/ethanol		6–150	[40]	sonication/water	1–4	0.7–1.2	[22]
planetary mill/NMP	2	20	[41]	H_2_SO_4_−thermal exfoliation/DMF	15–25	6–8	[42]
sonication/NMP	1.5	1	[10]	sonication and stirring/water-PVA	1–2	0.7–2	[29]
attrition/ethanol	1–5	6–8	[43]	Hummers-sonication/ethanol	5	5	[44]
planetary mill/melamine	25	6–8	[16]	Hummers-sonication/water-hydrazine	2	1	[3]
sonication/IPA-PVP	4	10	[15]				
attrition/ethanol	1–25	10–25	[13]				

The variety in the graphene source and the quality of exfoliation, dispersion and mixing methods, however, accounts for the big differences in the microstructure of the processed materials and therefore in the measured properties. Platelets of different dimensions (thin, thick or mixed), and morphologies (flat, undulated, wrinkled), have been reported in most cases as homogeneously dispersed but start to agglomerate as the filler content increases. For these reasons, more complex routes for mixing graphene with the matrix, avoiding agglomerate formation, have been effectively used. For example, *in-situ* graphene growth [47,48], CVD graphene growth on ceramic fibres that are subsequently mixed with the matrix [49] and the infiltration of graphene foams [50].

**Figure 2.**
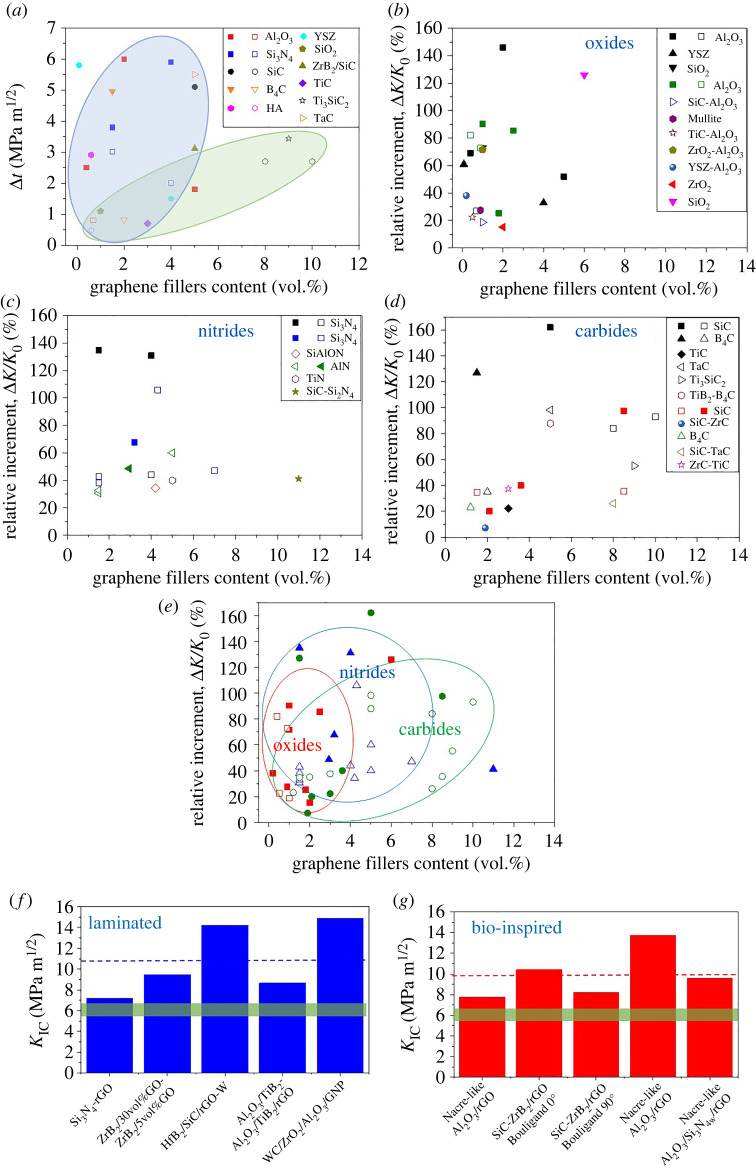
(*a*) Fracture toughness increments (Δ*K*_IC_) as a function of graphene filler content (vol.-%) for different basic bulk ceramic composites reported before 2017. Blue and green ellipses indicate areas with majority of rGO reinforced composites and GNP reinforced composites, respectively. Adapted from [58], no permission required. Δ*K*_IC_/*K*_0_ reported for basic bulk composites in recent years, separated by matrix type (*b*) oxides [11,12,22,31,32,59–62], (*c*) nitrides [15–17,41,46,63–67] and (*d*) carbides [19–21,25,27,28,68–71]. Materials already reported in Figure 2*a* have been included in black colour. Filled and empty symbols correspond to rGO and GNP platelets, respectively. (*e*) Comparison of toughness enhancements between oxides (red), nitrides (blue) and carbides (green). Filled and empty symbols correspond to rGO and GNP platelets. Other structures developed in recent years (*f*) laminated composites [29,45,51,72,73] and (*g*) bioinspired composites [37,45,54,74]. Dashed lines mark the average *K*_IC_ obtained for each type of structure. The green bar indicates the average *K*_IC_ achieved for bulk composites. (Online version in colour.)

The excellent results in the reinforcement of basic bulk composites, alongside the interest in replicating natural materials structures and using novel fabrication methods like additive manufacturing, have motivated the study of the mechanical response in other designs of ceramics containing graphene fillers. Under these premises, fracture resistance has been studied also in laminated materials [35,45,51] (figure 1*b*), functionally graded materials [26,52], coatings [36,53] (figure 1*c*), bioinspired structures [37,54] (figure 1*d*), porous [55] and three-dimensionally printed scaffolds [56,57] (figure 2*e*).

## 3. Methods for measuring the fracture toughness

The investigation of the fracture toughness in ceramic/graphene composites has been carried out through different methods. Indentation fracture toughness (IFT) is the most frequently used due to its accessibility, small sample size required and rapidity. It has become an essential technique for the observation of crack patterns and for localizing the GNP and GO sheets within them with the aid of scanning electron microscopy (SEM), which often reveals a toughening mechanism. This has also allowed us to establish relationships between the dispersion, morphology and orientation of the platelets and the crack-stopping mechanisms. IFT however, though a highly used method for determination of *K*_IC_ in isotropic materials, is not recommended in the case of graphene reinforced ceramics as the anisotropy of the composites creates a complex stress distribution under the indentation, producing branched crack patterns instead of the one generated by radial cracks [14]. Nonetheless, due to the advantages mentioned before, it continues to be a recurrent technique for *K*_IC_ measurement and it can be valid for contents below 5 vol.-% [10], which reduces the possibility of microcracks formation.

An effort to perform more reliable studies has been made through the application of single edge notched beam (SENB) [41] or single edge V-notched beam (SEVNB) [37] methods, and following normalized procedures such as those described in ASTM C1421 [75], surface crack in flexure (SCF) [18] and Chevron-notched beam (CB) [10]. These methods consist of the fracture of a specimen with an artificial crack subjected to bending. Also, they present difficulties associated with the achievement of proper notches/pre-cracks/sample geometries and testing conditions, needing a large quantity of samples to yield enough valid tests. It is important to mention that as the composites containing graphene fillers present increased fracture toughness, it is possible to observe higher stability in crack propagation, but this condition would also depend on the method selected. SEVNB is widely used due to the facile notch cut, but notch radius should be comparable to the material's microstructure to avoid overestimation of *K*_IC_. CB is a good alternative for obtaining stable fracture tests but is limited by having special tools for cutting the Chevron notch. On the contrary, SCF presents the advantage of introducing an easy artificial flaw by indentation but post-fracture identification of the pre-crack is complicated and could be affected by high filler contents. These bending test configurations are especially convenient for composites containing highly exfoliated graphene sheets. Yihua *et al*. [20] compared the results of IFT to those of the SENB test in SiC/rGO composites finding a large difference of up to 70% in the measured values, though the tendency of the fracture toughness with filler content was similar.

Complementarily, the inspection of fracture surfaces from the two halves of the fractured beams has provided interesting information on the tortuosity of the crack propagation paths [23,33] and the aspect of the pulled-out platelets [19]. Dynamic tests such as nanodynamic mechanical analysis (NanoDMA test) to assess the damping behaviour of TaC/GNP ceramics [76] and more recently *in-situ* crack propagation on Si_3_N_4_/rGO samples inside SEM [77] has permitted the direct confirmation of non-conventional mechanism, intrinsic to the graphene platelets.

## 4. Results on toughness enhancement

When compared to one-dimensional fillers, e.g. CNT, the use of platelets as reinforcing phase in composite materials presents various advantages associated with a reduced production cost, easier mixing methods and large available surface for effective stress transfer and increased interfacial energy available. Therefore, graphene (and its derivatives) was considered from the beginning as the two-dimensional variant of CNT from which higher relative enhancement could be expected. Moreover, it was also predicted that the particular layered microstructure of the platelets combining the extraordinary stiffness of the *ab*-plane with a weak bonding in the *c*-axis could generate additional non-conventional mechanisms.

Figure 2*a* shows the tendency of the maximum increments in fracture toughness both achieved by GNP and rGO for materials studied before 2017 [6]. It is notable that the same type of ceramics showed different relative increments with graphene fillers additions which can be explained by the differences in raw materials (ceramic matrix grain size, type and dimensions of graphene platelets) and processing methods. It also illustrates that a higher relative increment in *K*_IC_ could be achieved by the incorporation of rGO. Such platelets, as has been summarized in table 1, exhibit thicknesses below 5 nm, functionalized surface and higher roughness. The functional groups that remain after sintering, in addition to the wrinkled and flexible nature, create more clamping sites with matrix than the flat, thicker GNP.

As the types of matrices used and the number of publications have increased over time, presently there is enough data to study this tendency in the different families of structural ceramics. Figure 2*b–d* depicts toughness increment for individual materials within the same family of ceramics. Besides, all the results have been included in figure 2*e* for a direct comparison between families. Oxide-based materials are characterized by the preferential use of GO sheets that, as it was indicated above, are suitable for colloidal processing and possess a defective surface which improves interfacial strength. For nitrides, both types of graphene platelets have been used, achieving the highest increments with rGO, and also using GNP of high aspect ratio obtained after specific milling conditions [16]. Nitrides and carbides processed for potential wear applications require a higher volume of graphene fillers, therefore more varied contents can be found.

The selection of ceramic matrices has also changed from one-component matrices to systems that include other ceramic phases in the form of dispersed particles or whiskers which enhance hardness, high temperature properties and oxidation resistance. For instance, the fracture toughness of a high entropy ceramic composed of four materials (HfC, ZrC, TaC and WC) has been also successfully enhanced by 70% with the use of 0.15 wt-% of GNP [78]. The maximum increments observed are similar for the three types of matrices with the highest measured *K*_IC_ in the range of approximately 7–10 MPam^1/2^. However, as can be observed in figure 2*f,g*, higher values can be achieved with complex bioinspired or layered designs that allow better control of anisotropy, larger layers and reduction of agglomerates, enhancing the activation of mechanisms for energy dissipation, and giving values above the average of bulk materials.

## 5. Reinforcing mechanisms

**Figure 3.**
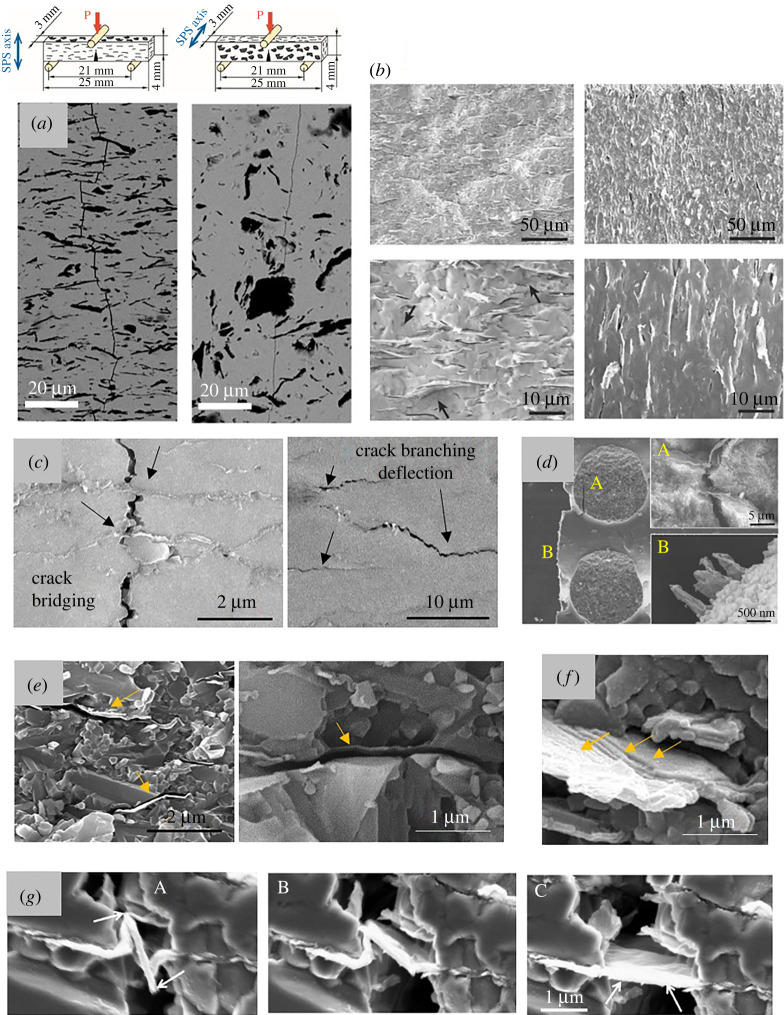
(*a*) Crack deflection in 8YSZ/GNP composites for two test configurations, with crack propagation path perpendicular to platelets plane, and with the crack front facing the edges of the platelets. (*b*) Fracture surfaces of specimens tested under the configurations described in (*a*). Notice the rough surface produced by a larger deflection of the crack path when platelets are aligned perpendicularly to it. Adapted from [23] with permission from Elsevier. (*c*) Crack branching and crack bridging produced by rGO platelets in Si_3_N_4_ composites. Images courtesy of GCT-ICV. (*d*) Bridging and pull-out of graphene oxide ribbons in three-dimensionally printed Al_2_O_3_ composite. [79] No permission required. (*e*) Post-fracture identification of the place occupied by pulled-out graphene platelets. Some external layers of the platelets remain bonded to the matrix. Images courtesy of GCT-ICV. (*f*) High magnification of a pulled-out platelet showing crinkles and sliding of internal layers. Image courtesy of GCT-ICV. (*g*) Sequence of images acquired during stable crack propagation test in Al_2_O_3_/rGO sample showing high capability of the bridging platelet for crinkling and stretching. Adapted from [80] with permission from Elsevier. (Online version in colour.)

The static and dynamic observations of crack propagation paths and fracture surfaces, accompanied by the results of *K*_IC_ measurements, confirm that both GNP and rGO act as suitable reinforcements in many types of ceramic matrices, clearly developing two types of mechanisms: (i) principal extrinsic mechanisms of conventional CMC reinforced by ligaments, which implicate the interaction between the matrix and the secondary phase, specifically crack deflection, bridging and pull-out (figure 3*a–e*) and (ii) mechanisms which involve how the graphene layers internally split and slide during fracture, namely the splitting of graphene individual layers, the bonding of external layers to the matrix allowing sliding of internal sheets during pull-out, and the kinking, bending and stretching of platelets (figure 3*e–g*).

Crack deflection occurs when a propagating crack tilts and twists, surrounding the platelets at the brittle interface formed with the matrix. Ovid'ko and Sheinerman have calculated that for a crack propagating at grain boundaries and encountering randomly oriented platelets *K*_IC_ could increase up to 50% [81], depending on platelet aspect ratio and volume fraction. This mechanism has been observed in test configurations where the crack propagation plane faces the graphene planes, which is the favourable condition to obtain the highest reinforcement, and the configuration commonly reported in the literature due to platelets alignment after sintering (left image in figure 3*a,b*). Moreover, brick-mortar biomimetic designs take advantage of this effect, forcing crack to propagate through the weak phase, achieving higher stability of the fracture process and more tortuous paths [74]. Other test configurations in which the crack propagation plane encounters the edges of the platelets have shown no significant deviation of crack paths [23] (right image in figure 3*a,b*). It is worth mentioning that interfaces with varied fracture properties could be formed between matrix and graphene due to the direct bonding between the layers and the ceramic grains or with the grain boundary phase composed by the sintering additives. Moreover, two situations seem to occur in the light of the type of platelets, (i) GNP platelets form weak bonds with the matrix allowing debonding at interface, activating deflection, and short bridging and pull-out. (ii) rGO platelets form strong bonding with the matrix which leads to improved stress transfer, debonding in between graphene planes, higher closure stresses and large pull-out distances.

The factors mentioned act in combination with residual stresses from thermal expansion mismatch and with the final structure of the platelets once the composite is sintered, as in many cases full exfoliation could not be achieved and big stacks formed by weakly bonded layers constitute the reinforcing ligament. In fact, Liu *et al.* have studied the interfacial strength between HA/graphene, observing that the highest load transfer could occur in the platelets' external layers [33]. In regions with strong matrix/platelet interfaces, which could imply a crack propagation through the platelet, the mechanism of deflection could remain active as the crack can still be driven in between the graphene planes. The importance of interface control in ceramic/graphene composites has been pointed out in recent years [82] and further investigations to determine the role of residual stresses in platelets debonding need to be carried out to optimize the reinforcing effect. The coefficient of thermal expansion (CTE) of structural ceramics is in the range approximately 3–10 × 10^−6^ K^−1^, lower than out of plane CTE of the platelets (27 × 10^−6^ K^−1^), which would produce tensile stresses perpendicular to the graphene plane; on the contrary, other conditions are expected at the edges of the platelets, affected by a lower in-plane CTE.

Crack wake bridging is the mechanism that contributes the most to fracture toughness enhancement by the generation of closing forces from the resistance of partially debonded graphene fillers and the mechanical interlocking with the matrix. An analysis using the model for fibre reinforced ceramics, taking experimental data from Si_3_N_4_ composites with aligned rGO and GNP platelets, estimated a value for graphene fillers strength in the order of 20–40 GPa [83], while the influence of volume fraction, platelets length and thickness on the toughness increment has been recently studied [84], also taking the results from YSZ/graphene composites, showing that longer and thinner platelets produce higher toughening ratio. Controlled crack propagation test in materials containing rGO sheets with varied dimensions have shown that short thin platelets exhibit more classical brittle fracture while thicker, larger stacks present more flexibility and stretching capability during pull-out [77]. Recently, molecular dynamics simulations of the pull-out mechanism of graphene platelets embedded in a SiC matrix have determined that bridging forces mainly contribute at the end of the sheets and there is only a slight increment in pull-out forces when platelet thickness changes from monolayer to multilayer graphene, but the conditions that favour toughening are more related to an increase in the volume of large FLG [85].

It is important to note that in addition to the differences observed in the relative increment of toughness related to the processing routes, it is commonly reported that toughness increment reaches a maximum at a specific filler volume and then drops gradually. The effect of higher graphene contents could lead to agglomeration and the formation of pores surrounding the platelets (or in between the agglomerate) which can contribute to diminishing the fracture toughness. Increased filler volume also augments the contact between platelets with different alignments which can contribute to rapid crack propagation creating easy paths between the weakly bonded planes.

## 6. R-curve, fatigue and mechanical properties at high temperature

Fewer works have been published addressing the study of R-curve behaviour, due to inconveniences related to the varied raw materials, the reproducibility of GNP and rGO dispersions, and the methods for performing stable crack propagation tests. Centeno *et al*. were the first to report rising R-curve behaviour in Al_2_O_3_/rGO composites obtaining 1.6 times higher steady-state toughness than initial *K*_0_ [86]. The achievement of sharp reproducible notches (1 µm radius) obtained by laser ablation in 8YSZ/GNP materials [23] allowed for the use of the compliance method for the comparison between composites with GNP contents up to 10.5 vol.-%, for the two configurations in which the graphene plane is perpendicular to the crack propagation plane (figure 3*a*). The results confirmed that the highest reinforcements are obtained for crack fronts facing platelets planes and the materials showed rapid increment in *K_R_* at short crack extensions when compared with other reported composites. Closure stresses of 50 MPa and a length of the bridged zone of 800 nm were calculated. A similar analysis of R-curve behaviour for two different orientations of the graphene platelets against crack propagation path, and for two morphologies of graphene platelets was performed in Al_2_O_3_/rGO materials by measurement of crack-opening displacements. The calculated maximum bridging stresses for thin and thick rGO sheets perpendicular to crack growth direction were 137 and 183 MPa, respectively [80]. The higher capability for dissipation of fracture energy of complex structures, compared with platelets homogeneously dispersed in a matrix, is reflected in the increasing crack resistances that have been achieved in brick-mortar ZrB_2_/SiC/rGO composites fabricated following different Bouligand patterns which exhibit *K_R_*/*K*_0_ ratios up to 2 (for the fibres with 45° rotation angle at crack lengths of 0.5 mm) [37].

Two interesting characteristics associated with cracks formation and propagation in graphene/ceramic composites that have been scarcely investigated are the behaviour of materials under cyclic stresses and the effect of temperature on fracture resistance. Fatigue in some monolithic ceramics may be difficult to assess as crack growth can be promoted by ambient conditions, and catastrophic failure may also occur instantly once the crack starts propagating, however, it has been studied in reinforced ceramics which exhibit more stable crack growth. For graphene/ceramic materials, the research is still at an early stage but a few works have shown important results. The damping behaviour of graphene deposited on Si/SiO_2_ substrate and in bulk TaC/GNP ceramics has been measured under quasi-static loading and low-frequency dynamic loading demonstrating an effective increase in damping and the possibility of reducing flaw formation by shock waves and vibrations through intrinsic energy dissipating mechanisms as rippling and flattening, bending, kinking and sliding [76,87]. Graphene deposited on Si_3_N_4_/polyethylene naphthalate substrate subjected to cyclic bending stress showed a fatigue limit two orders of magnitude higher than ITO deposited on the same substrate [88]. In a recent study, Wang *et al.* reported for the first time the observation of bridging rGO platelets crinkling and recovering flat morphology without apparent damage under the loading–unloading–reloading operations of a controlled crack propagation test, which was caused by mechanisms associated with weak Van der Waals forces, strain gradients and electromechanical coupling [80] (figure 3*c*).

The degradation of the graphene fillers and the mechanical integrity of the composites under high-temperature conditions are also relevant topics as an important part of the target applications for structural ceramics imply hot environments. Research on the stability of graphene at high temperature has been conducted in the range 600–1000 °C in argon during 1 h, simulating conventional sintering treatments. Under these conditions, it was seen that etching and vacancy defects were gradually induced [89]. In the case of high temperature SPS used for densification of ZrB_2_/graphene composites, an increment in D/G band ratio was observed by Raman spectroscopy, indicating an increase in defect concentration and the possible formation of ZrC [90]. Some observations in Al_2_O_3_ based materials have also associated the increment of structural defects in graphene platelet induced by high temperature sintering with a reduction of the toughening capacity [91].

Regarding the performance of the composites at elevated temperatures, Román-Manso *et al*. [92] carried out Hertzian indentation test at pre-creep temperatures up to 850°C, calculating the yield stress, toughness and resistance to cone/ring cracking of SiC/graphene composites obtained by *in-situ* growth. The three properties exhibited an important decrease above 400°C with more gradual decay for the composite with coarser microstructure. The creep resistance of ZrO_2_/GO ceramics has also been studied by uniaxial compression creep test at 1200–1250°C in argon, obtaining stress, grain size and apparent activation energy creep parameters. The creep resistance of monolithic ZrO_2_ dropped after graphene reinforcement which was explained by an increment in grain mobility of the composites produced by the lubricating character of the graphene filler [22].

## 7. Applications of graphene reinforced ceramics

Looking at the literature, especially in the last five years, it can be easily noticed that one of the main motivations for the incorporation of graphene fillers into ceramic composites respond to the enhancement of electrical performance, which can be exploited for fabricating novel products in the fields of electronics, sensing, energy harvesting and storage, and catalytic materials. The attractiveness of the development of these applications is attributed to the natural abundance of graphite source, graphene extraordinary electrical and thermal conductivity, high surface area and dimensionality, making it a second phase more than appropriate for mixing with nanomaterials and maximizing their properties. Extended information in reference to the multiple applications of ceramics containing graphene-based materials has been published recently in two reviews [58,93] describing key processing steps and relevant achievements for each field.

In the case of the structural materials considered in the present work, the addition of graphene fillers has two fundamental objectives: (i) the improvement of fracture resistance for making more damage-tolerant materials, intended to be used in parts subjected to high tensile stress, impact stress and wear, and (ii) the simultaneous improvement of the mechanical properties and the electrical or thermal functionalities of the composites which allow increasing the efficiency of the components in electronic devices or machining through alternative methods. The former includes, for example, Al_2_O_3_, Si_3_N_4_, SiC, WC, ZrC and TiB_2_ based ceramics developed for cutting tools, crucibles, automotive and aerospace components, armours, parts in nuclear reactors, and novel three-dimensionally printed prototypes, seeking to augment the reliability of products, reduce the losses from parts under friction and expand their life span. As examples of structural ceramics which take advantage of graphene electrical and thermal conductivities AlN, SiC, SiO_2_, TaC and ZrO_2_ based materials used in electronic packing and substrates, heat exchangers, and electromagnetic interference shields can be mentioned.

## 8. Conclusion

Huge progress has been made in the last decade taking advantage of graphene's extraordinary mechanical properties in crack initiation and arrest. The increased availability of graphene in larger quantities at lower cost has brought a wide variety of fillers with differences in size and surface functionalization, complicating the task of optimizing the conditions for structural reinforcement. The main achievements have been the demonstration of the reinforcing capability in different types of matrices and the identification of the mechanisms that reduce fracture energy. There is no unique approach to achieving a significant increment of fracture toughness, rather the characteristics of the matrix and the nature and distribution of the graphene sheets must also be considered. Additionally, in-depth studies on the role of defects and residual stresses are still needed for designing proper interfaces. The interesting results obtained from reinforcing ceramics with graphene have opened new fields of study focused on the use of other two-dimensional nanoreinforcements.

## Data Availability

Data are contained within the article.
