# Correction to ‘10 years of research on toughness enhancement of structural ceramics by graphene’ (2022) by Cristina Ramírez

**DOI:** 10.1098/rsta.2022.0299

**Published:** 2022-11-14

**Authors:** Cristina Ramírez

*Phil. Trans. R. Soc. A* **380**, 20220006. (Published online 1 August 2022). (doi:10.1098/rsta.2022.0006)

In the original version of this article, there was an error in the legend of figure 2*c*, where the chemical formula of silicon nitride was incorrectly provided as Si_2_N_4_ instead of Si_3_N_4_. This has now been corrected.

